# Prevalence of Popular Smoking Cessation Aids in England and Associations With Quit Success

**DOI:** 10.1001/jamanetworkopen.2024.54962

**Published:** 2025-01-17

**Authors:** Sarah E. Jackson, Jamie Brown, Vera Buss, Lion Shahab

**Affiliations:** 1 Department of Behavioural Science and Health, University College London, London, United Kingdom; 2 SPECTRUM Consortium, United Kingdom

## Abstract

**Importance:**

A wide range of medications, noncombustible nicotine products, behavioral support, and alternative treatments are available in England to help people stop smoking. Understanding their effectiveness outside of clinical trial settings can support informed decision-making.

**Objectives:**

To provide up-to-date estimates of the prevalence of different smoking cessation aids and associations with quit success and to explore moderation by socioeconomic position.

**Design, Setting, and Participants:**

This survey study used data from the Smoking Toolkit Study, a monthly cross-sectional household survey, conducted from 2006 to 2024 in England, UK. Participants were individuals aged at least 16 years who reported trying to quit smoking in the past year. Analyses were conducted from July to November 2024.

**Main Outcomes and Measures:**

The outcome variable was self-reported success in quitting smoking from the start of the most recent quit attempt up to the time of survey (hereafter, *quit success*). Independent variables were use (yes or no) of the following aids in the most recent attempt: nicotine replacement therapy (NRT), obtained via prescription or over-the-counter; varenicline; bupropion; electronic cigarettes (e-cigarettes); face-to-face behavioral support; telephone support; written self-help materials; websites; smartphone apps; hypnotherapy; Allen Carr’s Easyway method; heated tobacco products (HTPs); and nicotine pouches. Covariates included sociodemographic characteristics and features of the quit attempt.

**Results:**

A total of 25 094 participants (mean [SD] age, 38.7 [15.3] years; 51.5% [95% CI, 50.8%-52.2%] men) were included. In 2023 to 2024, the most used aids were e-cigarettes (40.2% [95% CI, 37.6%-42.8%]) and over-the-counter NRT (17.3% [95% CI, 15.3%-19.2%]); 40.8% (95% CI, 38.2%-43.4%) of quit attempts were unaided. While e-cigarette use was associated with higher odds of quit success after adjustment for use of other aids and covariates (odds ratio [OR], 1.95 [95% CI, 1.74-2.17]), use of over-the-counter NRT was not (OR, 1.03 [95% CI, 0.93-1.15]). Other aids positively associated with quit success were websites (used by 4.6% [95% CI, 3.5%-5.7%] in 2023-2024; OR, 1.43 [95% CI, 1.03-1.98]), prescription NRT (used by 4.5% [95% CI, 3.4%-5.5%] in 2023-2024; OR, 1.33 [95% CI, 1.12-1.58]), varenicline (used by 1.1% [95% CI, 0.5%-1.7%] in 2023-2024; OR, 1.80 [95% CI, 1.50-2.18]), and HTPs (used by 0.7% [95% CI, 0.3%-1.1%] in 2023-2024; OR, 2.37 [95% CI, 1.24-4.51]). Face-to-face behavioral support (used by 2.2% [95% CI, 1.5-2.9] in 2023-2024) was associated with higher odds of quit success among those from less advantaged (OR, 1.59 [95% CI, 1.19-2.14]) but not more advantaged (OR, 0.91 [95% CI, 0.65-1.29]) socioeconomic positions. There was not clear evidence of a benefit of any other aid, although some analyses were inconclusive.

**Conclusions and Relevance:**

This cross-sectional study found that while a range of effective smoking cessation aids are available in England, many people tried to quit using less effective forms of support or none at all. Quit success rates could be improved by encouraging people to use more effective methods.

## Key Points

**Question:**

What methods are people using to stop smoking in England and to what extent are they associated with quit success?

**Findings:**

This survey study including 25 094 smokers aged at least 16 years found that e-cigarettes were both the most commonly used cessation aid (used in 40.2% of quit attempts in 2023-2024) and associated with the highest odds of successful cessation. By contrast, other aids found to be associated with increased odds of success in quitting smoking were used in less than 5% of quit attempts.

**Meaning:**

These findings suggest that quit success rates could be improved by encouraging people to use more effective methods.

## Teaser

This survey study estimates the prevalences and effectiveness associated with different smoking cessation aids in England.

## Introduction

Stopping smoking is one of the best things a person can do to improve their health, but it can be very difficult.^1^ Nicotine delivered via cigarettes and other combustible tobacco products is highly addictive, and despite people’s best intentions, most quit attempts fail.^2^ Failure rates are typically higher among people from more disadvantaged socioeconomic groups,^3^ contributing to health inequalities.

A variety of aids are available to help people stop smoking, including medications (eg, nicotine receptor partial agonists), noncombustible nicotine products (eg, nicotine replacement therapy [NRT], electronic cigarettes [e-cigarettes]), behavioral support (eg, face-to-face, digital), and alternative treatments (eg, hypnotherapy). Many of these have been found to increase quit rates in randomized clinical trials (RCTs).^4,5,6,7,8^ However, trial results do not always replicate in clinical and home settings, so findings must be triangulated with observational evidence.^9^

In England, the best estimates of associations between different smoking cessation aids and quit success outside of RCTs come from the Smoking Toolkit Study, a nationally representative monthly cross-sectional survey gathering data on smoking and cessation since 2006.^10^ In a series of studies, we have analyzed data accumulated from an increasing sample of smokers to compare success rates of those trying to quit with different aids, adjusting for potential confounders (eg, level of addiction and other features of the quit attempt).^11,12,13,14,15,16^ The largest analysis, including 18 929 participants up to July 2018, suggested e-cigarettes and varenicline were the most effective methods: after adjustment for covariates and use of other aids, participants who used these in their most recent past-year quit attempt had 1.95 and 1.82 times higher odds, respectively, of quitting successfully than those who did not.^11^ Effectiveness of the assessed aids was largely consistent across socioeconomic positions, except websites, which were associated with better odds of quitting for disadvantaged smokers.^11^ However, results were insensitive for most other aids studied, meaning more data are required to draw firmer conclusions on their effectiveness.^11^

This study aimed to provide a comprehensive update on the associations between the use of smoking cessation aids in England and success in quitting smoking. Using data collected between 2006 and 2024, we updated our previous estimates for prescription NRT, NRT bought over the counter, varenicline, bupropion, e-cigarettes, face-to-face behavioral support, telephone support, written self-help materials, websites, and hypnotherapy. In addition, we extended the analysis to cover other types of behavioral support (smartphone apps and Allen Carr’s Easyway method [a pharmacotherapy-free behavioral program]) and newer noncombustible nicotine products (heated tobacco products and nicotine pouches). We also examined whether socioeconomic position moderated treatment effectiveness and provided data on the prevalence of use of each aid over time.

## Methods

This survey study was approved by the University College London Research Ethics Committee. Participants provide verbal informed consent to take part in the study, and all methods are carried out in accordance with relevant regulations. The study conformed to the American Association for Public Opinion Research (AAPOR) reporting guideline. The analysis plan was preregistered on Open Science Framework.^17^

### Design and Sample Selection

The Smoking Toolkit Study is an ongoing monthly cross-sectional household survey.^10^ It uses a hybrid of random probability and simple quota sampling to select a new representative sample of approximately 1700 individuals aged at least 16 years in England each month.^10^ Data were collected face-to-face up to the start of the COVID-19 pandemic and via telephone (both landline and mobile) since April 2020.^18^ Data were not collected from individuals aged 16 to 17 years between April 2020 and December 2021. We analyzed cross-sectional data collected in the period from November 2006 (the first wave of data collected) to June 2024 (the most recent data at the time of analysis). We selected participants who reported smoking cigarettes or any combustible tobacco product daily or occasionally in the past year (ie, past-year smokers) and having made at least 1 serious quit attempt in the past 12 months.

### Measures

Our outcome of interest was self-reported success in quitting smoking from the start of the most recent quit attempt up to the time of survey (hereafter, *quit success*). We assessed use of cessation aids as the exposure. Full details of the measures are provided in the eMethods in Supplement 1.

Independent variables were self-reported use or not (dummy coded) of the following smoking cessation aids in the most recent quit attempt: prescription NRT, NRT bought over the counter, varenicline, bupropion, e-cigarettes, face-to-face behavioral support, telephone support, written self-help materials, websites, smartphone apps, hypnotherapy, Allen Carr’s Easyway method (delivered face-to-face), heated tobacco products, and nicotine pouches. Participants indicated all that applied, with data coded 1 if chosen and 0 otherwise. This dummy-coding approach allowed analysis of the association between the use of specific aids and quit success while controlling for the use of other aids.

Some aids were not assessed in every wave: written self-help materials were included from March 2007, websites and Allen Carr’s Easyway from April 2008, e-cigarettes from July 2009, smartphone apps from February 2012, heated tobacco products from April 2016, and nicotine pouches from June 2021. We imputed missing values as 0 for participants surveyed before the response options were introduced, as these products were rarely used immediately after being added. Heated tobacco products were introduced to the market in November 2016^19^ and nicotine pouches around June 2019.^20,21^ Covariates included age, gender, socioeconomic position (indexed by an occupational measure and categorized as ABC1 [more advantaged] vs C2DE [less advantaged]^22^), level of addiction (assessed with a rating of strength of urges to smoke^23^), time since the quit attempt started, the number of prior quit attempts in the past year, whether the quit attempt was planned, whether the participant cut down first or stopped abruptly, calendar month, survey year, and mode of data collection.

### Statistical Analysis

We analyzed the data using R software version 4.2.1 (R Project for Statistical Computing), applying survey weights to match the demographic profile of adults in England.^10^ All analyses were done on complete cases. *P* values were 2-sided, and statistical significance was set at *P* < .05. Analyses were conducted from July to November 2024.

#### Preregistered Analysis: Associations With Quit Success

We calculated the quit success rate (with 95% CI) among users of each aid. Then we used logistic regression to analyze associations between quit success (abstinent vs not) and the use of different aids. We ran 3 models. Model 1 included all other aids (to estimate unique associations) but no covariates. Model 2 included covariates but no other aids. Model 3 was fully adjusted for all aids and covariates.

To test moderation by socioeconomic position, we repeated model 3 with interactions between aids and socioeconomic position in separate models. Where there was evidence of moderation of treatment effectiveness, we reran models 1 through 3 in stratified analyses.

We calculated planned Bayes factors (using an online calculator^24^) for nonsignificant findings to assess whether the data favored the null hypothesis or were insensitive.^25,26^ The specified effect was represented by a half-normal distribution reflecting an odds ratio (OR) of 1.5 (a conservative estimate in the ballpark of interventions known to be effective^27^).

#### Unplanned Analyses

We reported the proportion of participants using each aid. Because our study spanned a 17.5-year period, during which the availability and use of the various aids differed, we graphically displayed the proportions using each aid across the study period (calculated as 3-month moving means, to reduce noise) to provide context on changes over time. To provide up-to-date estimates of aid use, we separately reported the proportion of participants surveyed in 2023 to 2024 who reported using each aid.

Among individuals using at least 1 aid, we ran an additional logistic regression model to explore whether quit success differed between those using 1 vs 2 or more cessation aids, adjusting for covariates. For participants who used a nicotine product (e-cigarettes, NRT, heated tobacco products, or nicotine pouches) during their quit attempt and quit successfully, we estimated the proportion still using that product after cessation.

Finally, we assessed the population-level impacts of each aid on smoking cessation, taking into account their effectiveness and prevalence of use. We calculated impact scores as the prevalence of use of each aid in 2023 to 2024 multiplied by its association with effectiveness (fully adjusted OR – 1).

## Results

We analyzed data from 25 094 participants (weighted mean [SD] age, 38.7 [15.3] years; 51.5% [95% CI, 50.8%-52.2%] men). Across all eligible waves, data were collected from 77 372 past-year smokers, of whom 26 789 (34.6%) reported trying to quit in the past year. We excluded 1695 (6.3%) with missing data on 1 or more variables, leaving a final sample of 25 094 individuals. More missing data occurred after the switch from face-to-face to telephone interviews; however, the analyzed sample was similar to those who were excluded in their sociodemographic characteristics and quit attempt features (eTable 1 in Supplement 1).

### Prevalence of Use

More than half (55.8% [95% CI, 55.1%-56.5%]) of participants reported using at least 1 cessation aid in their most recent quit attempt (Table 1). Among this group, most (85.5%[95% CI, 84.8%-86.1%]) used a single aid, while 10.7% (95% CI, 10.1%-11.2%) used 2, and 3.9% (95% CI, 3.5%-4.2%) used 3 or more.

**Table 1.** Prevalence of Use of Cessation Aids in England, Across the Whole Study Period and in 2023-2024^a^

Cessation aid used in most recent quit attempt^b^	Whole study period (N = 25 094)		2023-2024, % (95% CI) (n = 1642)^d^
	No.^c^	% (95% CI)	
E-cigarettes^e^	4459	19.0 (18.4-19.5)	40.2 (37.6-42.8)
Over-the-counter NRT	6258	24.5 (23.9-25.1)	17.3 (15.3-19.2)
Websites^e^	430	1.9 (1.7-2.0)	4.6 (3.5-5.7)
Prescription NRT	1842	7.0 (6.6-7.3)	4.5 (3.4-5.5)
Smartphone apps^e^	186	0.8 (0.7-0.9)	3.6 (2.6-4.7)
Nicotine pouches^e^	84	0.4 (0.3-0.5)	3.1 (2.2-4.1)
Written self-help materials^e^	425	1.8 (1.6-2.0)	2.3 (1.5-3.0)
Face-to-face behavioral support	1013	3.8 (3.6-4.1)	2.2 (1.5-2.9)
Telephone support	224	0.9 (0.8-1.0)	1.3 (0.8-1.9)
Hypnotherapy	213	0.9 (0.7-1.0)	1.2 (0.7-1.8)
Varenicline	1208	4.8 (4.5-5.1)	1.1 (0.5-1.7)
Bupropion	346	1.3 (1.2-1.5)	0.9 (0.4-1.5)
Heated tobacco products^e^	72	0.3 (0.2-0.3)	0.7 (0.3-1.1)
Allen Carr’s Easyway^e^	45	0.2 (0.1-0.3)	0.5 (0.1-0.9)
None of these	11 079	44.2 (43.5-44.9)	40.8 (38.2-43.4)

Abbreviations: e-cigarette, electronic cigarette; NRT, nicotine replacement therapy.

^a^ Corresponding data stratified by socioeconomic position are provided in eTable 2 in Supplement 1.

^b^ Not mutually exclusive; sorted from the most to least popular in 2023-2024.

^c^ Unweighted number of participants who reported using each aid.

^d^ Up-to-date estimates of prevalence of the use of each aid among participants surveyed between January 2023 and June 2024.

^e^ Use of written self-help materials was assessed from March 2007, websites and Allen Carr’s Easyway from April 2008, e-cigarettes from July 2009, smartphone apps from February 2012, heated tobacco products from April 2016, and nicotine pouches from June 2021; use was imputed as 0 before this.

Across the period, the most commonly used aids were over-the-counter NRT (24.5% [95% CI, 23.9%-25.1%]) and e-cigarettes (19.0% [95% CI, 18.4%-19.5%]), followed by prescription NRT (7.0% [95% CI, 6.6%-7.3%]), varenicline (4.8% [95% CI, 4.5%-5.1%]), and face-to-face behavioral support (3.8% [95% CI, 3.6%-4.1%]) (Table 1). Among users of multiple aids, the most popular combination was over-the-counter NRT and e-cigarettes (28.1% [95% CI, 26.0%-30.4%]), followed by over-the-counter NRT and behavioral support (17.1% [95% CI, 15.4%-18.9%]), over-the-counter NRT and prescription NRT (13.6% [95% CI, 12.0%-15.3%]), and prescription NRT and behavioral support (12.5% [95% CI, 11.0%-14.1%]). Of participants who used written self-help materials, 49.7% (95% CI, 44.6%-54.8%) also used at least 1 other aid.

**Figure 1.**
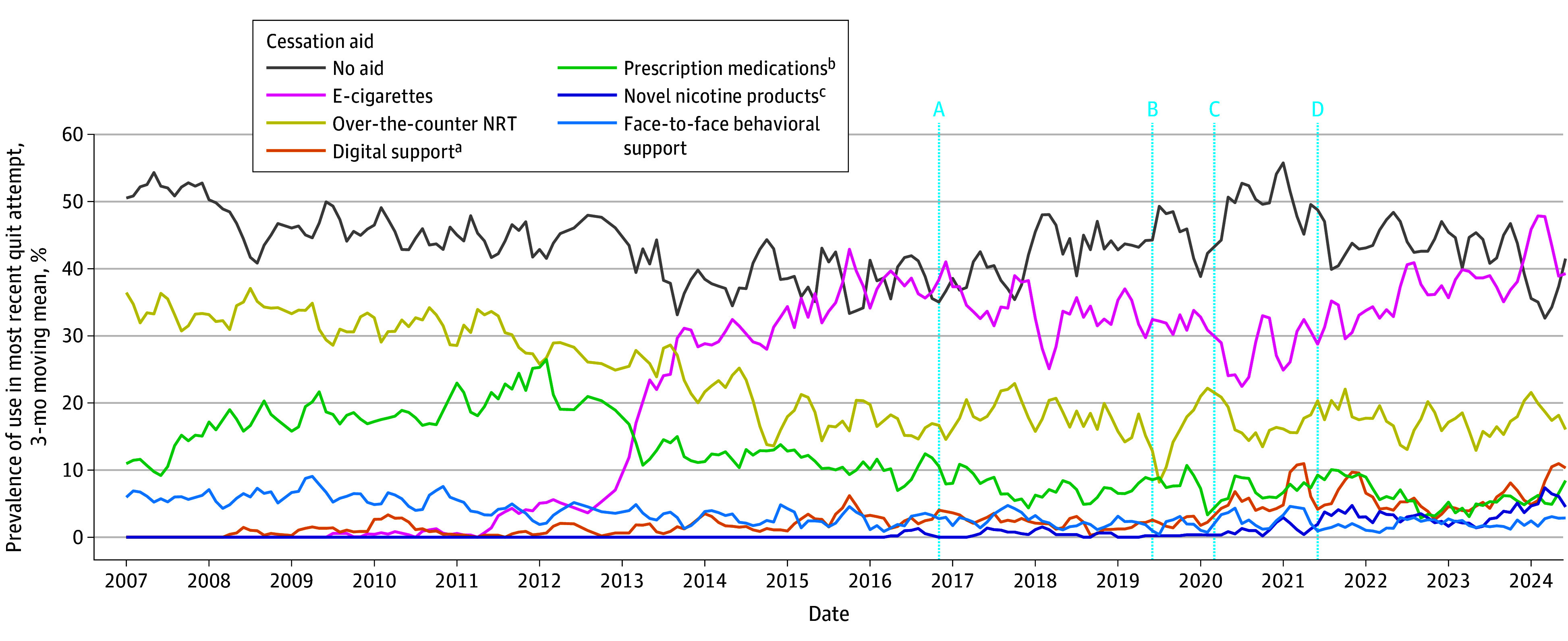
Monthly Prevalence of the Use of Smoking Cessation Aids in Quit Attempts in England Vertical lines indicate the timing of (A) the launch of heated tobacco products in November 2016, (B) the launch of nicotine pouches in July 2019, (C) the start of the COVID-19 pandemic in March 2020, and (D) the start of the varenicline supply disruption in England in June 2021. NRT indicates nicotine replacement therapy. ^a^Digital support includes websites and smartphone apps. ^b^Prescription medications include prescription NRT, varenicline, and bupropion. ^c^Novel nicotine products include heated tobacco products and nicotine pouches. Corresponding figures showing data separately for each of these aids, and other aids not presented here (written self-help materials, telephone support, Allen Carr’s Easyway method, and hypnotherapy) are provided in the eFigure in Supplement 1.

Patterns of aid use changed over time (Figure 1; eFigure in Supplement 1). There was a large increase in the use of e-cigarettes, which became the most popular aid used in 2013. There were also smaller increases in the use of novel noncombustible nicotine products (in particular, nicotine pouches) and digital support (websites and smartphone apps) in more recent years. There were decreases in the use of over-the-counter NRT, prescription medications (varenicline, NRT, and bupropion), and face-to-face behavioral support.

As of 2023 to 2024, the most used aids were e-cigarettes (40.2% [95% CI, 37.6%-42.8%]) and over-the-counter NRT (17.3% [95% CI, 15.3%-19.2%]). Websites and smartphone apps were used in 4.6% (95% CI, 3.5%-5.7%) and 3.6% (95% CI, 2.6%-4.7%) of quit attempts, respectively. Nicotine pouches were used in 3.1% (95% CI, 2.2%-4.1%) of quit attempts, while heated tobacco products were used in 0.7% (95% CI, 0.3%-1.1%). Prescription NRT was used in 4.5% (95% CI, 3.4%-5.5%) of quit attempts, while other prescription medications varenicline and bupropion were used in 1.1% (95% CI, 0.5%-1.7%) and 0.9% (95% CI, 0.4%-1.5%) of quit attempts, respectively. Furthermore, 40.8% (95% CI, 38.2%-43.4%) of quit attempts were unaided.

Use of cessation aids was broadly similar by socioeconomic position, although there were small differences in the use of certain aids (eTable 2 in Supplement 1). Participants from less advantaged socioeconomic positions were more likely than those who were more advantaged to use prescription NRT (7.6% [95% CI, 7.2%-8.1%] vs 6.0% [95% CI, 5.5%-6.5%]) and less likely to use written self-help materials (1.5% [95% CI, 1.3%-1.7%] vs 2.2% [95% CI, 1.9%-2.5%]) or hypnotherapy (0.7% [95% CI, 0.5%-0.8%] vs 1.1% [95% CI, 0.9%-1.3%]). They were also less likely to use heated tobacco products in 2023 to 2024 (0.2% [95% CI, 0.0%-0.5%] vs 1.4% [95% CI, 0.6%-2.2%]).

### Associations With Quit Success

Overall, 17.7% (95% CI, 17.2%-18.2%) of participants reported success in quitting smoking from the start of their most recent quit attempt up to the time of the survey. While there was no lower limit to the duration of the successful quit attempt, most participants reported that their quit attempt started 1 to 6 months (46.8% [95% CI, 46.1%-47.5%]) or more than 6 months (37.4% [95% CI, 36.7%-38.1%]) ago (eTable 1 in Supplement 1).

Table 2 shows unadjusted self-reported quit rates and sequentially adjusted models testing associations between each cessation aid and quit success. Unadjusted quit rates were highest among users of nicotine pouches (30.1% [95% CI, 19.3%-40.8%]), followed by heated tobacco products (29.9% [95% CI, 18.3%-41.5%]), smartphone apps (28.0% [95% CI, 21.0%-34.9%]), e-cigarettes (23.9% [95% CI, 22.6%-25.3%]), and websites (23.4% [95% CI, 19.0%-27.9%]).

**Table 2.** Associations of Use of Cessation Aids With Successful Quitting

Cessation aid used^a^	Unadjusted quit rate, % (95% CI)	Quit success, OR (95% CI)			Interaction with social grade^f^	Bayes factor^g^	Population-level impact^h^
		Model 1^b^^,^^c^	Model 2^c^^,^^d^	Model 3^c^^,^^e^			
Nonprescription nicotine products							
E-cigarettes	23.9 (22.6 to 25.3)	1.51 (1.38 to 1.65)	1.90 (1.70 to 2.11)	1.95 (1.74 to 2.17)	0.97 (0.79 to 1.18)	NA	38.2
Over-the-counter NRT	13.5 (12.5 to 14.4)	0.70 (0.63 to 0.76)	0.96 (0.87 to 1.06)	1.03 (0.93 to 1.15)	0.92 (0.76 to 1.13)	0.22	0.52
Nicotine pouches	30.1 (19.3 to 40.8)	1.74 (1.03 to 2.95)	1.45 (0.84 to 2.52)	1.21 (0.70 to 2.07)	0.55 (0.18 to 1.63)	0.95	0.65^i^
Heated tobacco products	29.9 (18.3 to 41.5)	1.84 (1.06 to 3.23)	2.35 (1.25 to 4.42)	2.37 (1.24 to 4.51)	0.34 (0.09 to 1.36)	NA	0.96
Prescription medications							
Prescription NRT	15.0 (13.1 to 16.8)	0.82 (0.71 to 0.95)	1.33 (1.13 to 1.58)	1.33 (1.12 to 1.58)	0.85 (0.61 to 1.19)	NA	1.49
Varenicline	19.7 (17.2 to 22.2)	1.16 (0.99 to 1.37)	1.67 (1.39 to 2.00)	1.80 (1.50 to 2.18)	1.00 (0.70 to 1.44)	NA	0.88
Bupropion	11.0 (7.5 to 14.6)	0.59 (0.41 to 0.86)	1.14 (0.76 to 1.69)	1.11 (0.73 to 1.69)	1.23 (0.51 to 2.97)	0.68	0.10^i^
Behavioral support							
Websites	23.4 (19.0 to 27.9)	0.73 (0.54 to 0.98)	1.62 (1.20 to 2.19)	1.43 (1.03 to 1.98)	1.68 (0.91 to 3.09)	NA	1.98
Smartphone apps	28.0 (21.0 to 34.9)	1.53 (1.06 to 2.20)	1.41 (0.92 to 2.16)	1.10 (0.71 to 1.71)	1.30 (0.56 to 2.98)	0.67	0.36^i^
Written self-help materials	14.0 (10.5 to 17.4)	1.31 (1.00 to 1.70)	0.76 (0.55 to 1.04)	0.73 (0.53 to 1.00)	0.93 (0.50 to 1.74)	0.13	−0.62
Face-to-face behavioral support							
Overall	16.7 (14.2 to 19.3)	1.04 (0.86 to 1.26)	1.38 (1.11 to 1.71)	1.26 (1.01 to 1.58)	1.62 (1.05 to 2.50)	NA	0.57
Social grades ABC1	15.9 (12.1 to 19.8)	0.81 (0.60 to 1.09)	1.05 (0.76 to 1.45)	0.91 (0.65 to 1.29)	NA	0.28	NA
Social grades C2DE	17.3 (13.8 to 20.7)	1.26 (0.98 to 1.62)	1.72 (1.30 to 2.27)	1.59 (1.19 to 2.14)	NA	NA	NA
Telephone support	17.0 (11.4 to 22.6)	0.99 (0.65 to 1.49)	1.23 (0.79 to 1.93)	0.93 (0.58 to 1.50)	0.44 (0.17 to 1.14)	0.42	−0.09^i^
Allen Carr’s Easyway	13.1 (2.7 to 23.5)	0.68 (0.27 to 1.73)	0.82 (0.25 to 2.73)	0.73 (0.20 to 2.70)	0.64 (0.05 to 9.07)	0.71	−0.14^i^
Alternative treatments							
Hypnotherapy	14.5 (9.6 to 19.4)	0.80 (0.53 to 1.20)	0.81 (0.54 to 1.21)	0.79 (0.52 to 1.22)	1.91 (0.86 to 4.24)	0.26	−0.25^i^
None of these	18.0 (17.3 to 18.8)	NA	0.67 (0.62 to 0.73)	NA	NA	NA	NA

Abbreviations: e-cigarette, electronic cigarette; NA, not applicable; NRT, nicotine replacement therapy; OR, odds ratio.

^a^ Sorted within categories from the most to least popular in 2023-2024.

^b^ Adjusted for use of all other cessation aids, but no covariates.

^c^ Model did not include the interaction term between cessation aid use and social grade.

^d^ Adjusted for covariates but no other cessation aids.

^e^ Fully adjusted for all cessation aids plus covariates.

^f^ Higher ORs indicate greater effectiveness (and lower ORs indicate lower effectiveness) of the smoking cessation aid among those from less advantaged social grades (C2DE) compared with those from more advantaged social grades (ABC1).

^g^ Bayes factors for non-significant findings in model 3, based on an expected OR of 1.5. Values 0.33 or less support the null hypothesis (ie, no difference in abstinence between use and non-use of the aid) and values between 0.33 and 3 suggest the data are insensitive.

^h^ Population-level impact of each aid on smoking cessation was calculated as prevalence of use in 2023 to 2024 multiplied by its association with effectiveness in this study (model 3 OR – 1). Higher values indicate greater population-level impact.

^i^ Inconclusive, based on Bayes factor results.

Analyses that adjusted for use of other cessation aids, but no covariates (model 1) indicated that smokers who used heated tobacco products, nicotine pouches, smartphone apps, e-cigarettes, and written self-help materials in their most recent quit attempt had higher odds of quit success than those who did not use these cessation aids (Table 2). Those who used bupropion, over-the-counter NRT, websites, and prescription NRT had lower odds of quit success. Odds were similar for users vs nonusers of varenicline, face-to-face behavioral support, telephone support, Allen Carr’s Easyway method, and hypnotherapy.

After adjustment for sociodemographic variables, level of addiction, factors relating to the quit attempt, month and year of the survey, and mode of data collection but excluding adjustment for other cessation aids (model 2), the odds of quit success were higher among those who used heated tobacco products, e-cigarettes, varenicline, websites, face-to-face behavioral support, or prescription NRT, and lower among those who tried to quit unaided (Table 2). A similar pattern of results was observed when use of other cessation aids was adjusted for (model 3) (Table 2).

**Figure 2.**
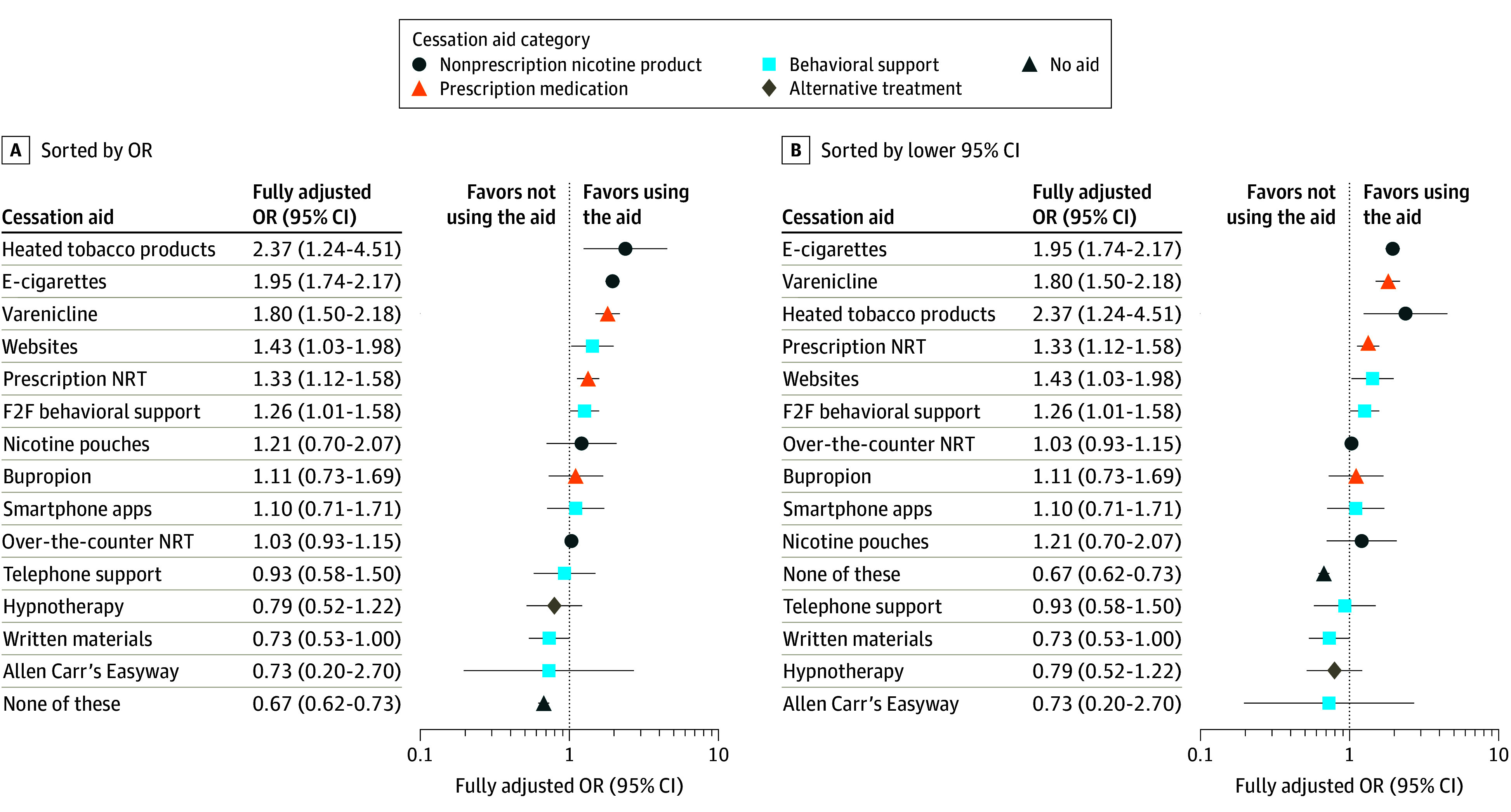
Fully Adjusted Associations Between Use of Cessation Aids and Quit Success Data shown are the results of fully adjusted logistic regression models testing the association between use of a given aid and success in quitting smoking. Results for each individual aid are adjusted for use of all other cessation aids plus covariates (model 3). Results for unaided quitting (ie, none of these) are adjusted for covariates (model 2). Error bars represent 95% CIs. F2F indicates face-to-face; NRT, nicotine replacement therapy; OR, odds ratio.

Figure 2 displays the fully adjusted results (model 3), sorted by the odds ratio (OR) and by the lower 95% CI. In each case, the 3 aids associated with the highest odds of quit success were e-cigarettes (OR, 1.95 [95% CI, 1.74-2.17]), varenicline (OR, 1.80 [95% CI, 1.50-2.18]), and heated tobacco products (OR, 2.37 [95% CI, 1.24-4.51]). While heated tobacco products had the largest OR, it also had the widest 95% CI (on account of the small number of participants using this aid; n = 72), meaning the size of the association was less certain. Positive associations were more modest for websites (OR, 1.43 [95% CI, 1.03-1.98]), prescription NRT (OR, 1.33 [95% CI, 1.12-1.58]), and face-to-face behavioral support (OR, 1.26 [95% CI, 1.01-1.58]). There was little evidence that the effectiveness of the different cessation aids (fully adjusted for use of other aids and covariates) differed by socioeconomic position (Table 2). For example, use of over-the-counter NRT was not associated with quit success (OR, 1.03 [95% CI, 0.93-1.15]). The only exception was use of face-to-face behavioral support, which was associated with higher odds of quit success among those from less advantaged socioeconomic positions (C2DE; fully adjusted OR, 1.59 [95% CI, 1.19-2.14]) but similar odds of quit success compared with nonuse among those from more advantaged positions (ABC1; fully adjusted OR, 0.91 [95% CI, 0.65-1.29]) (Table 2).

Bayes factors based on results from the fully adjusted model (Table 2) indicated that there was evidence to suggest there was no association between quit success and use (vs nonuse) of over-the-counter NRT, face-to-face behavioral support for smokers from more advantaged social grades, written self-help materials, and hypnotherapy. The data were insensitive to detect associations between quit success and use of nicotine pouches, bupropion, smartphone apps, telephone support, and Allen Carr’s Easyway method.

Among participants who reported using at least 1 cessation aid, unadjusted quit rates were 17.1% (95% CI, 16.4%-17.8%) for individuals who used 1 aid and 19.0% (95% CI, 17.1%-20.9%) for those who used 2 or more aids. After adjusting for covariates, those who used 2 or more aids had higher odds of quit success (OR, 1.32 [95% CI, 1.13-1.54]).

### Continued Nicotine Use After Smoking Cessation

Among participants who reported using an e-cigarette in their quit attempt and quit successfully, 84.8% (95% CI, 82.2%-87.5%) were still using e-cigarettes at the time of the survey. The corresponding figures for those who used NRT (either bought over-the-counter or obtained on prescription), heated tobacco products, and nicotine pouches in their quit attempt and who were still using this product at the time of the survey were 38.8% (95% CI, 35.4%-42.4%), 40.6% (95% CI, 8.7%-72.5%), and 24.4% (95% CI, 0.0%-52.6%), respectively.

### Population-Level Impact

We calculated an impact score for each aid taking into account the prevalence of use of each cessation aid in 2023 to 2024 and estimates of their effectiveness. These scores suggested that e-cigarettes had by far the greatest impact on smoking cessation at the population level (impact score, 38.2), followed by websites (impact score, 1.98), prescription NRT (impact score, 1.49), and heated tobacco products (impact score, 0.96) (Table 2).

## Discussion

This survey study examined the prevalence of use of different smoking cessation aids in England and associations with quit success. We found that many people who tried to stop smoking from 2006 to 2024 did so without using any support. This is the least effective way to quit: we found that after adjusting for their level of addiction and other features of their quit attempt, smokers who tried to quit unaided had around one-third lower odds of successfully quitting than those who used some form of support.

As of 2023 to 2024, the most commonly used smoking cessation aid was e-cigarettes, used in 40% of quit attempts. We found that quit attempts aided by e-cigarettes were more likely to be successful than those that were not. This is consistent with evidence from randomized clinical trials^5,28^ and previous observational studies^11,13^ and provides further evidence that, in addition to being popular, e-cigarettes offer one of the most effective methods of quitting smoking. Of individuals who used e-cigarettes to quit, most (85%) were still vaping at the time of the survey. This is in line with UK guidance, which encourages people not to rush to stop vaping immediately after quitting smoking, but to gradually decrease their vaping frequency or nicotine strength when they feel confident they can do so without risking relapse to smoking.^29^

The next most popular aid in 2023 to 2024 was over-the-counter NRT, used in 17% of quit attempts. While substantial trial evidence shows NRT to be an effective treatment^4^ (albeit less effective than e-cigarettes^5^), our data did not show any benefit of using over-the-counter NRT. We only found NRT to be associated with higher odds of success when it was obtained on prescription (which occurred much less frequently). This may be because when people buy NRT themselves, without any advice on how to use it effectively, they either do not use enough NRT or use it incorrectly.^30,31^ Evidence shows NRT is more effective for smoking cessation when used as a combination of a slow-release patch to suppress withdrawal symptoms and a fast-acting form (eg, gum, lozenge, inhaler) to satisfy in-the-moment urges.^8,32^

Other aids positively associated with success in quitting were heated tobacco products, varenicline, websites, and face-to-face behavioral support, but these were used much less frequently (<5% of quit attempts in 2023-2024). Of these, the effect estimate was largest for heated tobacco products, which were used in less than 1% of quit attempts in 2023 to 2024, but the 95% CI was wide on account of the small number of participants who reported using them. It will be important to update this analysis when more data are available to improve the precision of our estimate.

Varenicline, which we found to be another of the most effective smoking cessation treatments, has not been available in England for several years. Its supply was paused in July 2021 after its manufacturer, Pfizer, detected higher than acceptable levels of nitrosamine impurities in the drug.^33^ The shortfall in varenicline use has not been offset by increases in the use of other prescription medications (NRT and bupropion).^33^ Even if it had, these medications appear less effective for helping people quit, so they do not provide a like-for-like replacement.^8^ A generic version of varenicline has been available in some countries and was launched in England in late 2024. In addition, cytisine, a similar compound to varenicline started to be supplied on prescription via smoking cessation services from January 2024. Trial evidence suggests it is likely to be similarly effective to varenicline and e-cigarettes.^8^ We have been collecting data in the Smoking Toolkit Study on use of cytisine in quit attempts since April 2022 (prior to 2024, it was possible to buy it online or bring it into the UK from overseas) but there are not yet sufficient numbers of participants using cytisine to provide an estimate of its effectiveness in this population. We aim to look at this in future when the sample size allows.

Websites and smartphone apps have become more popular as quitting aids since 2020. This increase in the use of digital support coincided with the onset of the COVID-19 pandemic and may have been the result of people being less able to access in-person support^34,35^ or less willing to go to pharmacies or shops to buy products, such as NRT or e-cigarettes. In our previous analysis,^11^ which included data up to 2018, we found use of websites was associated with greater odds of quitting successfully among smokers from less (but not more) advantaged socioeconomic positions. The present analysis showed a different pattern: increased odds of quitting among people who used websites, with no evidence to suggest this association differed by socioeconomic position. This may potentially reflect changes in the profile of smokers using websites to quit in more recent years (as prevalence has increased), or improvements in the quality of information and support provided by the websites people are using.

This study is the first time we have examined the effectiveness of smartphone apps. Our results did not show a clear benefit: while app use was associated with increased odds of success in partially adjusted models, the association was much smaller when we adjusted both for covariates and for use of other aids. Trial evidence on apps is also inconclusive.^36,37,38^ It is likely effectiveness varies according to apps’ content^39^ and the extent to which they use evidence-based behavior change techniques.

Use of face-to-face behavioral support was associated with greater quit success among individuals from less advantaged socioeconomic positions, but there was no association among those who were more advantaged. This was the only aid for which we observed a clear moderating effect of socioeconomic position. In England, this type of support is typically delivered by free-to-use, local authority–commissioned stop-smoking services. These usually offer a combination of behavioral support and pharmacotherapy (eg, varenicline, NRT, bupropion, or in some services, e-cigarettes); any benefits of the pharmacotherapy provided are accounted for in our models by adjustment for use of other aids. The difference in effectiveness by socioeconomic position may indicate that pharmacotherapy is sufficient for helping more advantaged smokers to stop smoking, but that for those who are less advantaged, face-to-face behavioral support adds value in terms of further increasing quit rates. Qualitative research with smokers of different socioeconomic positions who have used stop-smoking services may be useful to gain insight into the elements people find most helpful.

There was not clear evidence of a benefit of any other aid, but some analyses were inconclusive. Although our overall sample size was large, some aids were used rarely across the entire period (bupropion, telephone support, and Allen Carr’s Easyway method) and some only started to become more popular late in the period (smartphone apps and nicotine pouches). As a result, there were only small numbers of users of these aids in our sample, which were not sufficient to draw firm conclusions. There is currently little evidence on the effectiveness of nicotine pouches,^40^ although their popularity appears to be increasing quickly. Evidence on smartphone apps is also limited and findings are mixed.^36,37,38^ However, trials suggest bupropion, telephone support, and Allen Carr’s Easyway method increase quit rates under controlled trial conditions.^8,41,42,43,44^ Bayes factors indicated our data were insensitive to detect benefits of these 5 aids, meaning we need to collect more data to determine how effective they are in natural settings. Our findings were clearer for written self-help materials and hypnotherapy: the data suggested using these aids was not associated with increased success in quitting. The lack of a clear benefit of written self-help materials contradicts trial evidence that shows that when no other support is available, using written self-help materials can increase quit rates.^45^ In our sample, approximately one-half of individuals who used written self-help materials also used at least 1 other aid, which may account for some of the difference in results.

### Strengths and Limitations

Strengths of this study include the representative sample, assessment of a wide range of smoking cessation aids, and adjustment for important confounders (eg, level of addiction). There were also limitations. Our outcome was based on self-reports of success in quitting smoking, with no fixed duration required to determine success, so the definition of successful quitting varied depending on how long ago participants’ most recent past-year quit attempt started. This could potentially have caused us to overestimate success rates associated with aids that have become more popular very recently (eg, nicotine pouches), if quit attempts involving these aids occurred closer to the time of the survey. The survey did not regularly capture the duration, frequency, intensity, or dosage of aids used or distinguish between different types of NRT. Use of some aids was not assessed consistently across the entire period, so values were imputed with 0 in waves before data were collected. However, this is unlikely to have had a substantial impact on the results, given the aids that were introduced later in the period had very low prevalence before we started assessing them. Although our overall sample was large, analyses of some aids that were used more rarely were limited by small samples. Therefore, this is an analysis that will need to be updated again when more data are available. Furthermore, we only considered the effectiveness of aids for cessation and not other factors that may be important to consider when making decisions about which aid to use in a quit attempt (eg, potential harms).

## Conclusions

In this survey study of smokers in England, we found that while a range of effective smoking cessation aids are available, many people tried to quit either using less effective forms of support or none at all. Quit success rates could be improved by encouraging people to use more effective methods.

## Notes

Supplement 1. **eMethods.** Full Details of Measures **eTable 1.** Weighted Sample Characteristics **eTable 2.** Prevalence of Use of Cessation Aids in England, Across the Whole Study Period and in 2023-2024 **eFigure.** Monthly Prevalence of the Use of Smoking Cessation Aids in Quit Attempts in England

Supplement 2.
Data Sharing Statement
