# Supplementary material for: Prevalence of Popular Smoking Cessation Aids in England and Associations With Quit Success
Source: JAMA Netw Open. 2025 Jan 17;8(1):e2454962. doi: 10.1001/jamanetworkopen.2024.54962 (PMC11742533; doi:10.1001/jamanetworkopen.2024.54962)
Supplement: Supplement 2. — Data Sharing Statement [file jamanetwopen-e2454962-s002.pdf]

Data Sharing Statement

Jackson. Prevalence and Effectiveness of Popular Smoking Cessation Aids in England. *JAMA Netw Open*. Published January 17, 2025. doi:10.1001/jamanetworkopen.2024.54962

Data
Data available: Yes
Data types: Deidentified participant data
How to access data: Open Science Framework (https://osf.io/uyw5k/)
When available: With publication

Supporting Documents
Document types: None

Additional Information
Who can access the data: anyone requesting the data
Types of analyses: to replicate our results
Mechanisms of data availability: without investigator support
Any additional restrictions: not to be used for other publications
